# Spatiotemporal Epidemiological Trends of Mpox in Mainland China: Spatiotemporal Ecological Comparison Study

**DOI:** 10.2196/57807

**Published:** 2024-06-19

**Authors:** Shuli Ma, Jie Ge, Lei Qin, Xiaoting Chen, Linlin Du, Yanbo Qi, Li Bai, Yunfeng Han, Zhiping Xie, Jiaxin Chen, Yuehui Jia

**Affiliations:** 1 School of Public Health Qiqihar Medical University Qiqihar China; 2 Scientific Research Office Qiqihar Medical University Qiqihar China

**Keywords:** mpox, spatiotemporal analysis, emergencies, prevention and control, public health

## Abstract

**Background:**

The World Health Organization declared mpox an international public health emergency. Since January 1, 2022, China has been ranked among the top 10 countries most affected by the mpox outbreak globally. However, there is a lack of spatial epidemiological studies on mpox, which are crucial for accurately mapping the spatial distribution and clustering of the disease.

**Objective:**

This study aims to provide geographically accurate visual evidence to determine priority areas for mpox prevention and control.

**Methods:**

Locally confirmed mpox cases were collected between June and November 2023 from 31 provinces of mainland China excluding Taiwan, Macao, and Hong Kong. Spatiotemporal epidemiological analyses, including spatial autocorrelation and regression analyses, were conducted to identify the spatiotemporal characteristics and clustering patterns of mpox attack rate and its spatial relationship with sociodemographic and socioeconomic factors.

**Results:**

From June to November 2023, a total of 1610 locally confirmed mpox cases were reported in 30 provinces in mainland China, resulting in an attack rate of 11.40 per 10 million people. Global spatial autocorrelation analysis showed that in July (Moran *I*=0.0938; *P*=.08), August (Moran *I*=0.1276; *P*=.08), and September (Moran *I*=0.0934; *P*=.07), the attack rates of mpox exhibited a clustered pattern and positive spatial autocorrelation. The Getis-Ord Gi^*^ statistics identified hot spots of mpox attack rates in Beijing, Tianjin, Shanghai, Jiangsu, and Hainan. Beijing and Tianjin were consistent hot spots from June to October. No cold spots with low mpox attack rates were detected by the Getis-Ord Gi^*^ statistics. Local Moran *I* statistics identified a high-high (HH) clustering of mpox attack rates in Guangdong, Beijing, and Tianjin. Guangdong province consistently exhibited HH clustering from June to November, while Beijing and Tianjin were identified as HH clusters from July to September. Low-low clusters were mainly located in Inner Mongolia, Xinjiang, Xizang, Qinghai, and Gansu. Ordinary least squares regression models showed that the cumulative mpox attack rates were significantly and positively associated with the proportion of the urban population (*t_0.05/2,1_*=2.4041 *P*=.02), per capita gross domestic product (*t_0.05/2,1_*=2.6955; *P*=.01), per capita disposable income (*t_0.05/2,1_*=2.8303; *P*=.008), per capita consumption expenditure (PCCE; *t_0.05/2,1_*=2.7452; *P*=.01), and PCCE for health care (*t_0.05/2,1_*=2.5924; *P*=.01). The geographically weighted regression models indicated a positive association and spatial heterogeneity between cumulative mpox attack rates and the proportion of the urban population, per capita gross domestic product, per capita disposable income, and PCCE, with high *R*^2^ values in north and northeast China.

**Conclusions:**

Hot spots and HH clustering of mpox attack rates identified by local spatial autocorrelation analysis should be considered key areas for precision prevention and control of mpox. Specifically, Guangdong, Beijing, and Tianjin provinces should be prioritized for mpox prevention and control. These findings provide geographically precise and visualized evidence to assist in identifying key areas for targeted prevention and control.

## Introduction

Mpox is a zoonotic viral disease caused by the mpox virus, primarily circulating in animals but also transmissible to humans [1,2]. The main reservoir of the mpox virus is believed to be rodents and primates such as squirrels, kangaroos, dormice, monkeys, and apes [3,4]. Mpox can be transmitted to humans through direct contact with infected animals, their bodily fluids, or contaminated materials [4]. While human-to-human transmission of mpox is less common, it can occur through respiratory droplets, close physical contact, or contact with skin lesions or bodily fluids of an infected individual [4]. An ongoing outbreak of mpox since January 1, 2022, has primarily affected men who have sex with men in countries outside of West and Central Africa, with cases reported to the World Health Organization (WHO) [5].

Historically, mpox has been mainly observed in Central and West Africa, with mortality rates ranging from 1% to 10% [6,7]. Since 2022, person-to-person transmission of mpox has been significantly increasing, thereby resulting in its widespread occurrence in other countries [8-10]. As of the end of February 2024, 94,707 confirmed cases of mpox, including 181 deaths, have been reported to the WHO from 117 countries and regions worldwide since January 1, 2022 [5]. In September 2022, the first imported case of mpox was reported in Chongqing, China. In June 2023, a local mpox epidemic occurred in China. As of November 30, 2023, 1611 confirmed cases of mpox have been reported from 30 (96.8%) provinces in mainland China, except Xizang, which included 1610 local cases [11].

The WHO declared mpox an international public health emergency on July 23, 2022 [12], which was reaffirmed on February 15, 2023 [13]. A public health emergency of international concern is described in the International Health Regulations (2005) as an extraordinary event that poses a public health risk to other countries through the international spread of a disease, potentially requiring a coordinated global response [14]. On September 15, 2023, mpox was classified as a category B infectious disease for management by the National Health Commission of China, effective September 20, 2023 [15]. In accordance with the Law of Infectious Disease Prevention and Control of the People’s Republic of China, statutory infectious diseases are identified by their transmission, epidemic intensity, and hazard degree. These diseases are classified into 3 categories, totaling 40 types: 2 types in category A, 27 types in category B, and 11 types in category C. Category A infectious diseases are considered compulsory for management, while category B infectious diseases are strictly regulated [16]. According to the WHO, the confirmation of a single case of mpox in a country is considered an outbreak [5]. Therefore, it is crucial to prioritize the mpox pandemic owing to its potentially disastrous consequences for public health, socioeconomic factors, and overall health care systems. Experiences from previous pandemics, such as SARS, Middle East respiratory syndrome coronavirus, and COVID-19, highlight the roles of health administrators and policymakers to promptly develop comprehensive prevention and control strategies in all countries.

Spatial epidemiological studies have accurately visualized the spatial distribution and clustering of diseases via mapping [17-20]. Using geographically precise and visual evidence, priorities for disease prevention and control could be identified, and their effectiveness could be evaluated [21-24]. Various factors can influence the occurrence and prevalence of infectious diseases: natural factors (climate and geography) and social factors (economy and population density). The existing evidence emphasizes the spatial heterogeneity in mpox distribution, indicating complexity and unevenness in the spatial patterns of mpox cases [25-27]. This highlights the significance of conducting spatial analyses in mpox studies. However, there is a scarcity of reports on spatial analyses of mpox.

The attack rate is a crucial indicator for measuring the frequency and intensity of disease occurrence and for evaluating the effectiveness of disease prevention and control within a short period. Therefore, we conducted a spatial epidemiological analysis to comprehensively and accurately describe and analyze the mpox attack rate. Our analysis aimed to identify spatial distribution characteristics and spatial clustering patterns and to determine the presence of cold and hot spots of spatial clustering. Furthermore, we examined the spatial regression relationship between the mpox attack rate and sociodemographic and socioeconomic factors.

## Methods

### Study Design

A spatial ecological comparison study was designed to conduct a spatiotemporal epidemiological analysis of mpox attack rates in mainland China at a provincial level, focusing on spatiotemporal distribution characteristics and clustering patterns of the mpox attack rate and its spatial relationship with sociodemographic and socioeconomic factors.

### Study Area

This study was conducted in 31 provinces of mainland China, excluding Taiwan, Macao, and Hong Kong (Table S1 in Multimedia Appendix 1). According to the Chinese administrative division, the 31 provinces are divided into 6 regions: north China, northeast China, east China, central south China, southwest China, and northwest China. As of the end of 2022, the estimated population of China was 1411.75 million people. The per capita gross domestic product (PCGDP) of China was US $12,720, and the per capita disposable income (PCDI) was US $5472. These data are sourced from the China Statistical Yearbook (2023) of the National Bureau of Statistics [28]. For a visual representation of the study areas and administrative divisions, please refer to Figure S1 in Multimedia Appendix 2.

### Study Population

This study analyzed all the locally confirmed cases of mpox in mainland China that occurred between June and November 2023. As of November 30, 2023, a total of 1610 locally confirmed cases of mpox have been reported by the National Health Commission of China [11].

### Mpox Attack Rate

The provincial-level crude mpox attack rates per 10 million people were calculated by dividing the total number of confirmed mpox cases in each province by its total population. This rate was multiplied by 10 million to obtain the rate per 10 million people.

### Sociodemographic and Socioeconomic Variables

Sociodemographic variables included the proportion of the urban population (PUP), natural population growth rate (NPGR), percentage of the illiterate population older than 15 years (PIP), aging rate older than 65 years (AR), and per capita road area (PCRA) [28]. Socioeconomic variables included the PCGDP, PCDI, per capita consumption expenditure (PCCE), and PCCE for health care (PCCEH) [28].

### Spatial Analysis

Spatial description and analysis were conducted using ArcGIS (version 9.0; Environmental Systems Research Institute, Inc), with the province as the spatial analysis unit. Thematic maps were created to visually and intuitively display the spatial distribution of the confirmed cases and attack rates of mpox.

Global spatial autocorrelation analysis was conducted to probe the presence of spatial clustering in mpox attack rates at a broader level [22]. This analysis used the global Moran *I* statistic (–1 ≤ values ≤ +1). Spatial autocorrelation was considered to be present if the *P* value was below .10, with a test level *α* of .10. The values of Moran *I* indicate whether the spatial distribution of the mpox attack rates was random (Moran *I*=0), dispersed (Moran *I*<0), or clustered (Moran *I*>0). However, it is important to note that global spatial autocorrelation analysis does not provide information on specific local locations and patterns of spatial clustering.

To accurately identify the geographic locations and patterns of spatial clustering at the provincial level of mpox attack rates within the study area, a local spatial autocorrelation analysis was conducted. The analysis used Getis-Ord-Gi^*^ and local Moran *I* statistics, which are commonly used spatial techniques for assessing local spatial autocorrelation [22]. The Getis-Ord Gi^*^ statistics identified 2 types of clustering: hot spots and cold spots. Hot spots are identified by positive *z* values, indicating that high values of mpox attack rates are clustered among neighboring provinces, while cold spots are identified by negative *z* values, indicating that low values of mpox attack rates are clustered among neighboring provinces. The corresponding *z* values for the Getis-Ord Gi^*^ statistic at 90%, 95%, and 99% CIs are ±1.65, ±1.96, and ±2.58, respectively. The local Moran *I* statistic was used to verify and complement the Getis-Ord Gi^*^ analysis, as it allows the detection of areas where spatial outliers exist. The results of the local Moran *I* highlighted aspects that may have been overlooked in the Getis-Ord Gi^*^ analysis. Local Moran *I* examines 4 patterns of spatial clustering: high-high (HH) clustering (positive correlation; high values of mpox attack rates clustered among neighboring provinces), high-low (HL) outlier (negative correlation; provinces with high values of mpox attack rates surrounded by those with low values), low-high (LH) outlier (negative correlation; provinces with low values surrounded by those with high values), and low-low clustering (positive correlation; low values clustered among neighboring provinces).

This study used ordinary least squares (OLS) and geographically weighted regression (GWR) models to examine the spatial regression relationship between the mpox attack rates and sociodemographic and socioeconomic factors. The dependent variable was the cumulative mpox attack rate between June 1 and November 30, 2023, and sociodemographic and socioeconomic factors were considered independent variables. The OLS model was used to estimate the global parameters, whereas the GWR model was used to estimate the local parameters, considering the spatial autocorrelation and spatial heterogeneity of the research factors [29,30]. The GWR model was constructed using the weighted least squares method, with the selection criterion and kernel function playing essential roles. The selection criterion determined the adaptive bandwidth size, while the kernel function calculated the weight matrix. This study used the fixed Gaussian and Akaike information criterion as the kernel function and selection criterion, respectively [30].

### Ethical Considerations

This study was approved by the ethical committee of Qiqihar Medical University (approval number [2021] 31). This study adhered to the Helsinki Declaration.

## Results

### Spatiotemporal Distribution of Confirmed Cases and Attack Rates of Mpox

**Figure 1:**
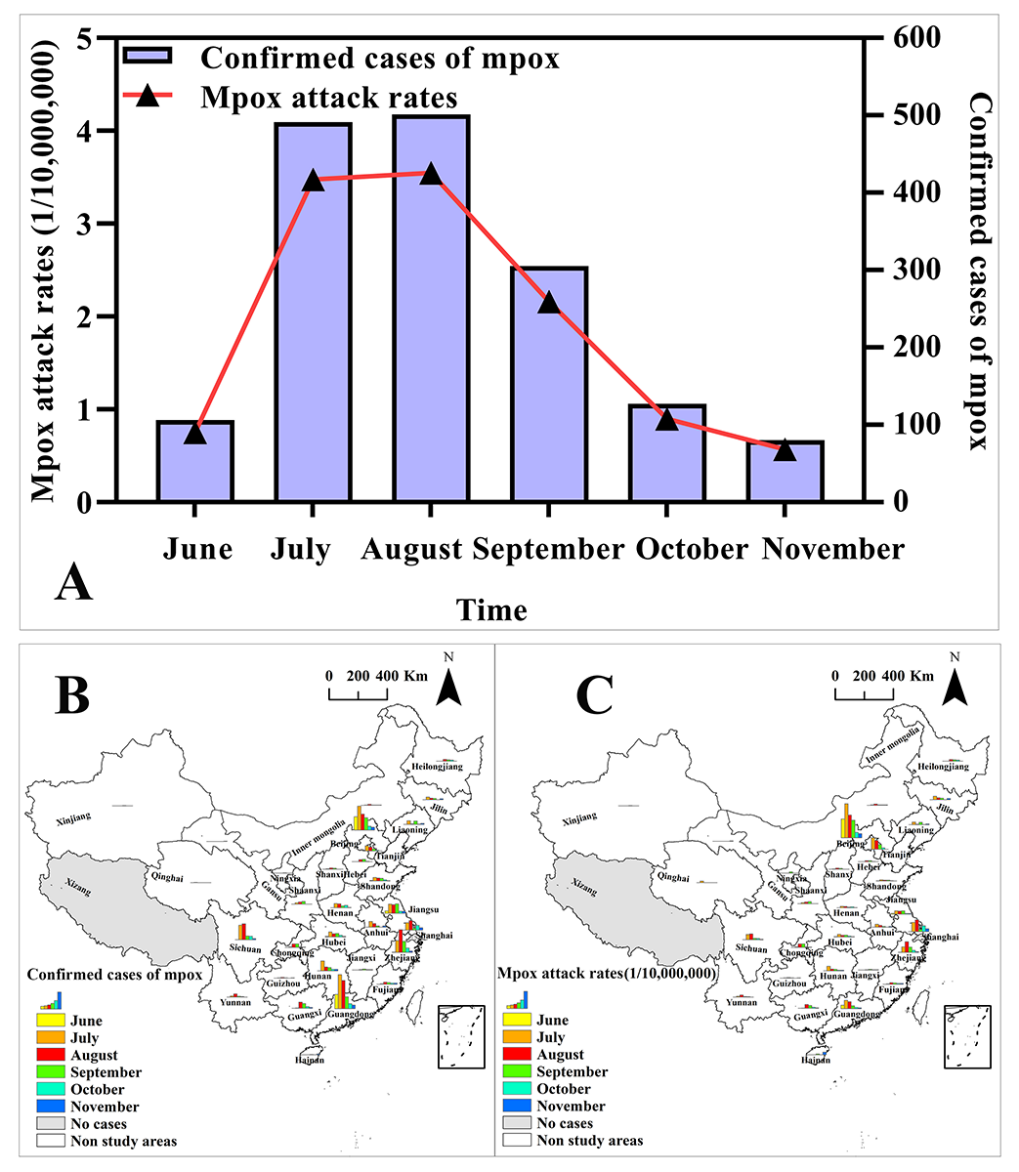
Spatiotemporal distribution of confirmed cases and attack rates of mpox. (A) Temporal distribution of confirmed cases and attack rates of mpox; (B) Spatiotemporal distribution of confirmed cases of mpox; (C) Spatiotemporal distribution of attack rates of mpox.

Between June 1 and November 30, 2023, a total of 1610 locally confirmed cases of mpox were reported in mainland China, resulting in an attack rate of 11.40 per 10 million people. The number of confirmed cases and the attack rate of mpox initially increased and then decreased during the study period, as depicted in Figure 1A-C. The peak period was observed between July and August, which accounted for 61.6% (n=992) of the total cases. In August 2023, mpox reached its highest level with 501 confirmed cases and an attack rate of 3.55 per 10 million people. However, by November, the number of confirmed cases and attack rate reached their lowest points and were even lower than the attack rate in June. There were 80 confirmed cases with an attack rate of 0.57 per 10 million people (Figure 1A).

**Figure 2:**
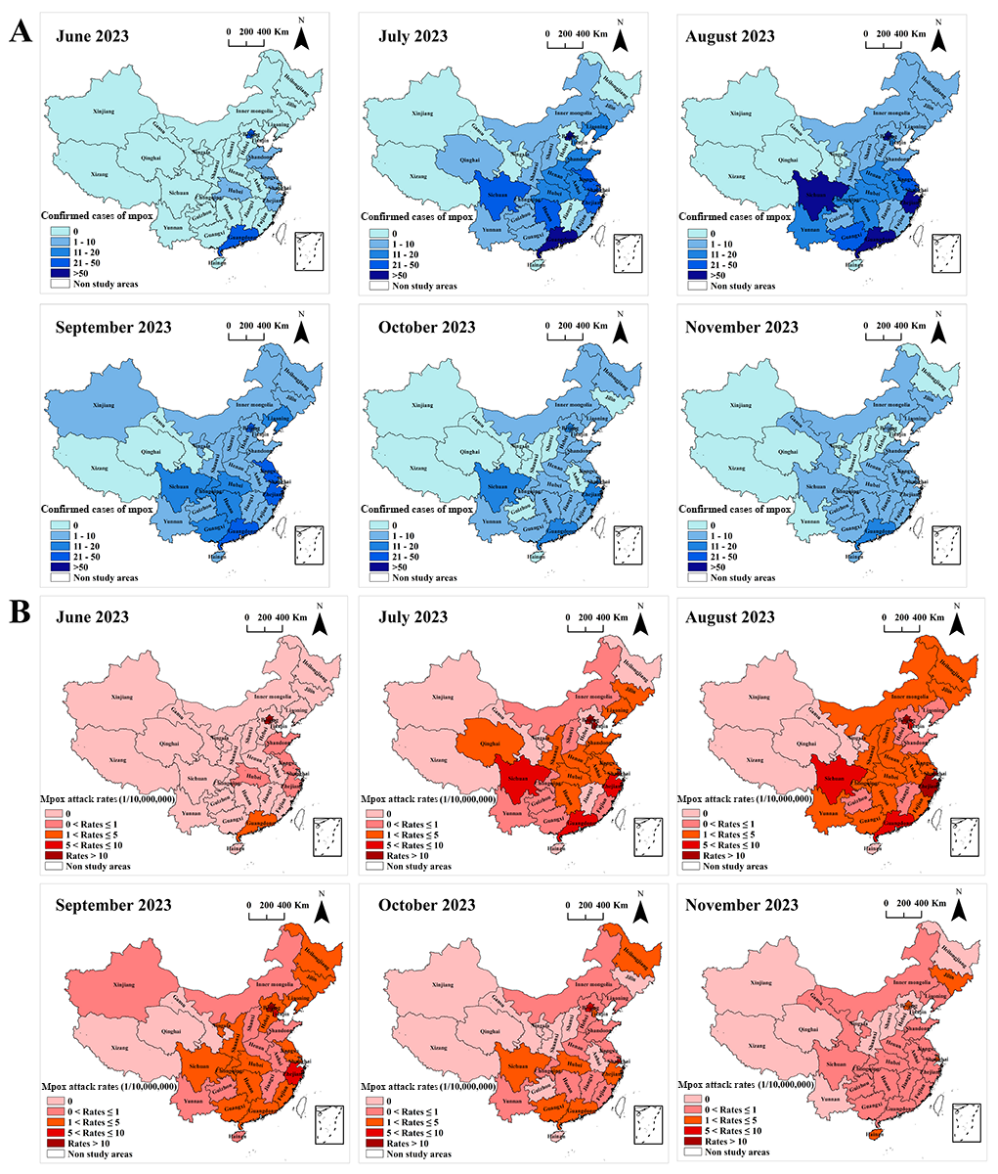
Spatiotemporal distribution of confirmed cases (A) and attack rates (B) of mpox from June to November 2023. A higher resolution version of this figure is available in Multimedia Appendix 4.

Between June 1 and November 30, 2023, a total of 30 provinces reported confirmed cases of mpox, with Xizang being the only province without any cases (Table S2 in Multimedia Appendix 1 and Figure S2 in Multimedia Appendix 3). Among these provinces, Guangdong, Beijing, Zhejiang, Sichuan, and Jiangsu had the highest cumulative numbers of confirmed mpox cases, with 327, 245, 176, 132, and 114 cases, respectively (Table S2 in Multimedia Appendix 1 and Figure S2A in Multimedia Appendix 3). Furthermore, the highest cumulative attack rates of mpox were observed in Beijing, Shanghai, Tianjin, Zhejiang, and Guangdong provinces, with rates of 112.18, 35.15, 28.61, 26.76, and 25.84 per 10 million people, respectively (Table S2 in Multimedia Appendix 1 and Figure S2B in Multimedia Appendix 3). The number of regions reporting confirmed mpox cases gradually increased between June and September 2023 (Figure 2A,B). Confirmed mpox cases were reported in 6 provinces in June, whereas in September, the number of regions reporting confirmed mpox cases rose to 28. However, from October to November 2023, there was a gradual decline in the number of regions reporting mpox cases (Figure 2A,B). Beijing consistently exhibited the highest mpox attack rates (Figure 2B). During July and August 2023, the number of confirmed cases and attack rates of mpox in various regions were particularly elevated, especially in Beijing, Guangdong, Shanghai, Sichuan, Tianjin, and Zhejiang provinces, when compared with other regions and months (Figure 2A,B).

### Global Spatial Autocorrelation Analysis of Mpox Attack Rates

**Figure 3:**
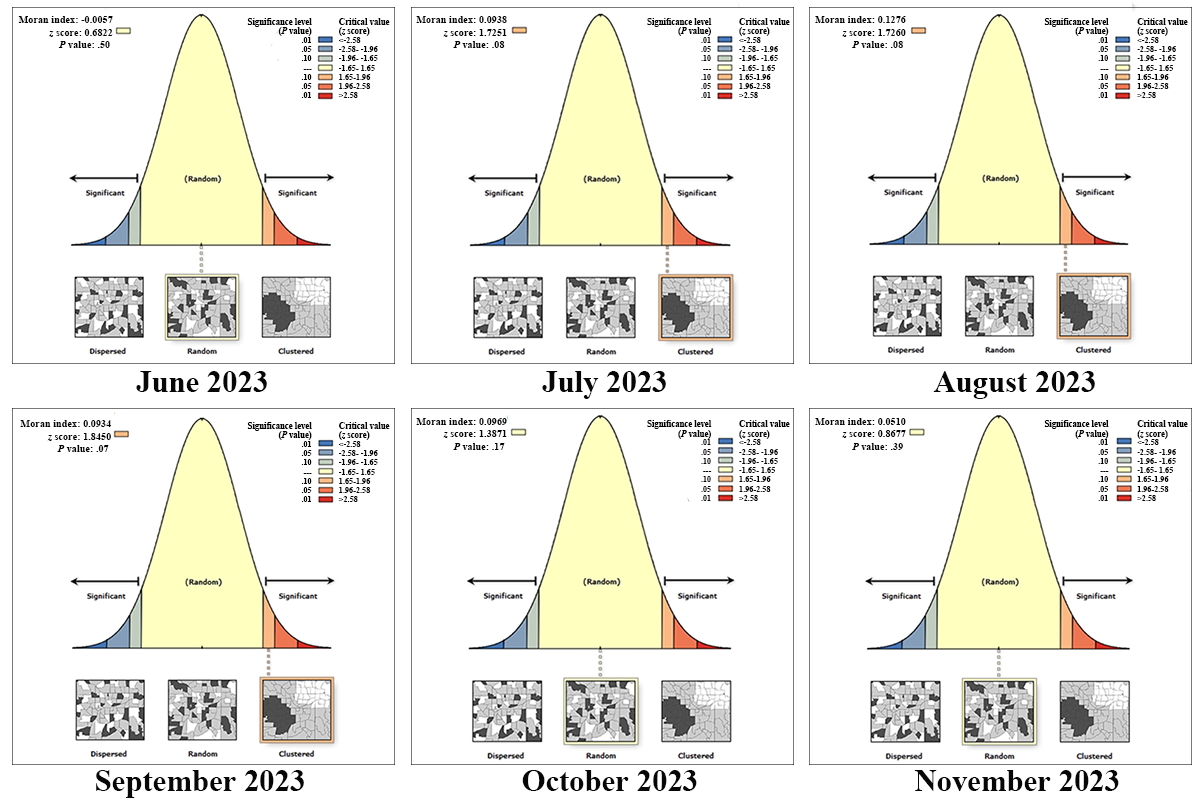
Results of Moran *I* statistic for global spatial autocorrelation analysis of mpox attack rates.

A global spatial autocorrelation analysis was conducted to examine the spatial patterns of mpox attack rates at the provincial level in mainland China (Table S3 in Multimedia Appendix 1). The results showed that in June (Moran *I*=–0.0057, *P*=.50), October (Moran *I*=0.0969, *P*=.17), and November (Moran *I*=0.0510, *P*=.39), the attack rates of mpox were randomly distributed and not statistically significant. However, there was a significant global spatial autocorrelation in July (Moran *I*=0.0938, *P*=.08), August (Moran *I*=0.1276, *P*=.08), and September (Moran *I*=0.0934, *P*=.07). This indicates that, during this period, the attack rates of mpox exhibited a clustered pattern and positive spatial autocorrelation (Figure 3).

### Local Spatial Autocorrelation Analysis of Mpox Attack Rates

**Figure 4:**
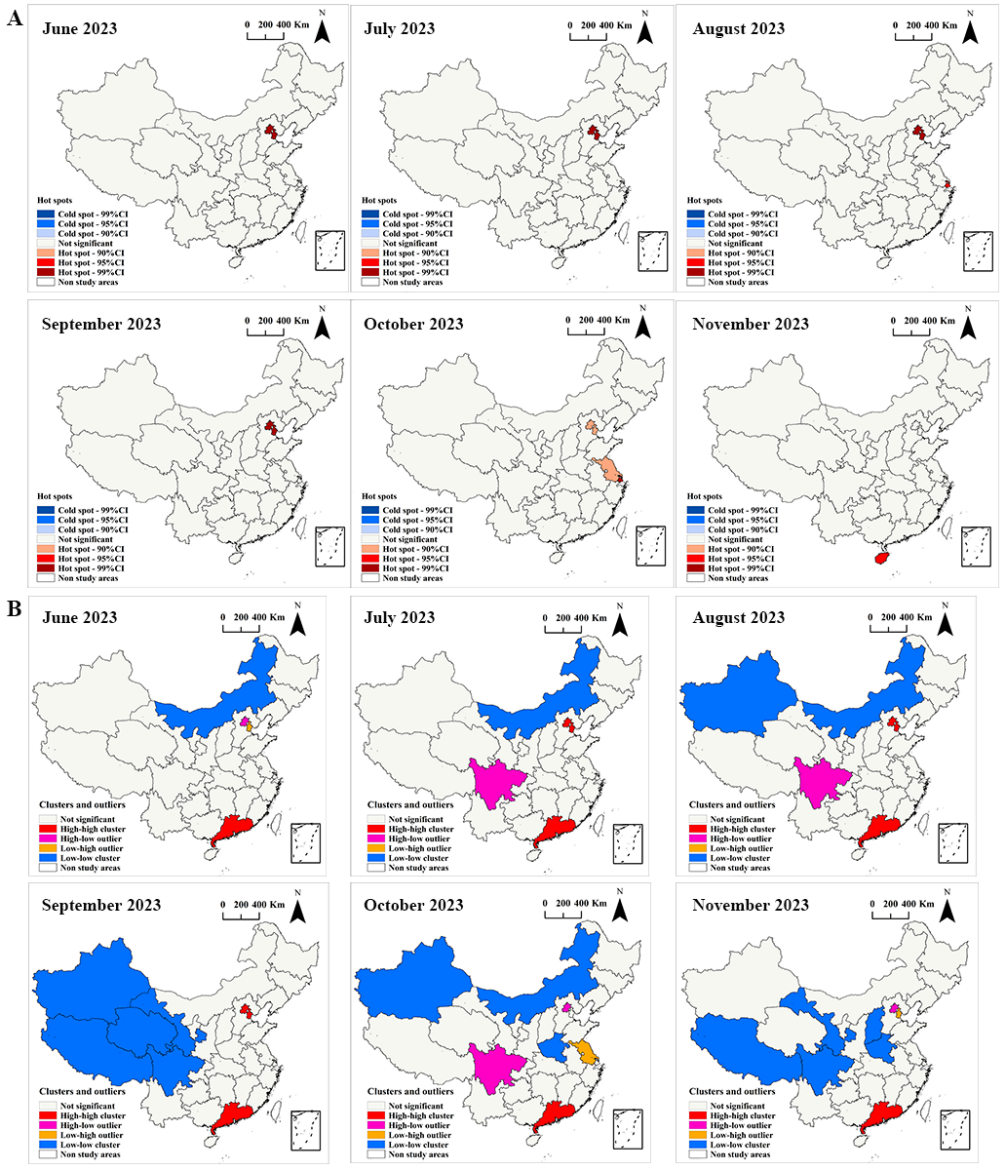
Results of local spatial autocorrelation analysis of mpox attack rates. (A) Getis-Ord Gi* statistic; (B) local Moran *I* statistic. A higher resolution version of this figure is available in Multimedia Appendix 5.

The Getis-Ord Gi^*^ statistics revealed that Beijing and Tianjin consistently exhibited high mpox attack rates from June to October, categorizing them as hot spots. Shanghai was identified as a hot spot in August and October. Jiangsu and Hainan were identified as hot spots in October and November, respectively. No cold spots with low mpox attack rates were detected using the Getis-Ord Gi^*^ statistics (Figure 4A).

According to the results of the local Moran *I* statistics, Guangdong province consistently exhibited HH clustering in terms of spatial patterns of mpox attack rates from June to November. Beijing and Tianjin were identified as HH clusters from July to September. Beijing was identified as an HL outlier for June, October, and November. Sichuan was determined to be an HL outlier in July, August, and October. Tianjin was identified as an LH outlier in June and November. Jiangsu was identified as an LH outlier in October. Low-low clusters were mainly located in Inner Mongolia, Xinjiang, Xizang, Qinghai, and Gansu (Figure 4B). This implies that areas with low mpox attack rates are clustered in neighboring regions.

### Spatial Regression Analysis Between Mpox Attack Rates and Sociodemographic and Socioeconomic Factors

The detailed values of the cumulative mpox attack rates and explanatory variables can be found in Tables S4 and S5 in Multimedia Appendix 1 and Figure S3 in Multimedia Appendix 6. The results of the Pearson correlation analysis indicated that the cumulative mpox attack rates were not significantly correlated with the NPGR, PIP, AR, and PCRA but were significantly and positively associated with the PUP, PCGDP, PCDI, PCCE, and PCCEH. However, there were significant correlations among the PUP, PCGDP, PCDI, PCCE, and PCCEH (Figure S4 in Multimedia Appendix 7). To address multicollinearity, separate OLS and GWR regression models were conducted for the dependent variable and each independent variable. However, multivariate OLS and GWR regression models have not yet been developed.

First, we used OLS regression models to explore the spatial regression relationship between the cumulative mpox attack rates from June 1 to November 30, 2023, as the dependent variable and sociodemographic and socioeconomic factors as independent variables. The cumulative mpox attack rates were not significantly correlated with the NPGR, PIP, AR, or PCRA; while they were significantly and positively associated with the PUP (*t_0.05/2,1_*=2.4041, *P*=.02), PCGDP (*t_0.05/2,1_*=2.6955, *P*=.01), PCDI (*t_0.05/2,1_*=2.8303, *P*=.008), PCCE (*t_0.05/2,1_*=2.7452, *P*=.01), and PCCEH (*t_0.05/2,1_*=2.5924, *P*=.01). The *R*^2^ values of the OLS models for the PUP, PCGDP, PCDI, PCCE, and PCCEH were 0.4190, 0.5743, 0.6469, 0.5583, and 0.5400, respectively; indicating that these 5 variables can explain the 41.90%, 57.43%, 64.69%, 55.83%, and 54% variance of cumulative mpox attack rates at the provincial level (Table S6 in Multimedia Appendix 1).

**Figure 5:**
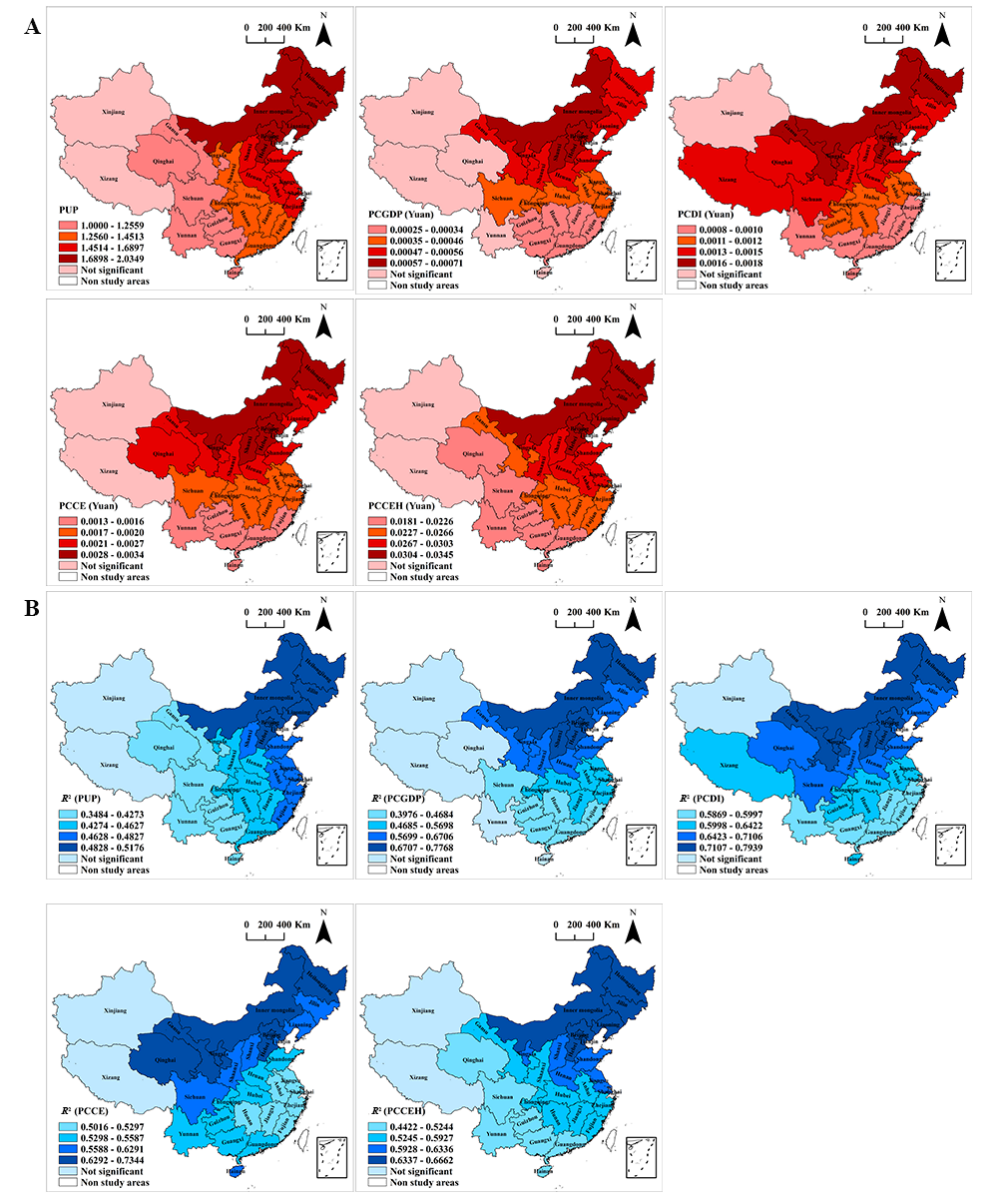
Spatial distribution of the coefficients (A) and local *R*^2^ (B) values of the explanatory variables in the geographically weighted regression model. PCCE: per capita consumption expenditure; PCCEH: PCCE for health care; PCDI: per capita disposable income; PCGDP: per capita gross domestic product; PUP: proportion of the urban population. A higher resolution version of this figure is available in Multimedia Appendix 8.

The GWR regression models were created using only the statistically significant independent variables identified in the OLS regression models, along with the cumulative mpox attack rates. The coefficients and local *R*^2^ values of the explanatory variables in the GWR models are presented in Figure 5 and Tables S7-S10 in Multimedia Appendix 1. Generally, the Akaike Information Criterion values of the GWR models were significantly lower than those of the OLS model, indicating that the GWR had a high explanatory power and better fitting ability (Tables S6 and S7 in Multimedia Appendix 1). The GWR model indicated a positive association and spatial heterogeneity between cumulative mpox attack rates and the PUP, PCGDP, PCDI, and PCCE (Figure 5), with high *R*^2^ values in north and northeast China.

## Discussion

### Principal Findings

This study is the first in mainland China to conduct a spatiotemporal epidemiological analysis to comprehensively identify the spatiotemporal characteristics and clustering patterns of confirmed cases and attack rates of mpox at the provincial level. The findings of this study are of great significance for improving the accuracy and effectiveness of mpox prevention and control.

Since June 2023, local cases of mpox have been reported in 30 of 31 provinces in mainland China, excluding Xizang [11]. In June, 6 provinces reported cases, which rose to 28 regions by September. However, from October to November 2023, there was a gradual decline in the number of regions reporting mpox cases. As of November 30, 2023, a total of 1610 locally confirmed cases of mpox have been reported [11]. The attack rate of mpox is 11.40 per 10 million. The number of confirmed cases and attack rate of mpox exhibited a pattern of increase from June to August, followed by a decrease from September to November. In November, both the number of confirmed cases and the attack rate reached their lowest points, even lower than the level observed in June. These findings highlight the importance of the implementation of preventive and control measures. On June 10, 2022, the National Health Commission of China formulated the “Diagnosis and Treatment Guidelines for Monkeypox (2022 edition)” [3]. Subsequently, on June 27, 2022, the National Health Commission of China released the Technical Guidelines for Monkeypox Prevention and Control (2022 edition) [4]. The guidelines highlight 4 key areas for action: first, enhancing the evaluation of the mpox pandemic situation; second, intensifying quarantine and monitoring measures for mpox; third, enhancing the professional training of health care workers; and finally, ensuring the availability of diagnostic reagents, therapeutic drugs, and vaccines for mpox [31]. On July 26, 2023, the National Disease Control and Prevention Administration developed the Monkeypox Prevention and Control Plan [32]. Subsequently, on August 2, 2023, the AIDS Prevention and Control Center of China created the Core Information of Monkeypox Prevention and Treatment for Key Populations, aiming to enhance awareness and prevention of mpox [33]. On September 15, 2023, mpox was classified as a category B infectious disease for management by the National Health Commission of China, effective September 20, 2023 [14]. These guidelines provide guidance on the diagnosis, treatment, prevention, and control of mpox in various regions, which also emphasizes the need to strengthen prevention and control measures for mpox.

Although the number of regions reporting mpox cases, confirmed cases, and attack rates declined from October to November 2023, the first confirmed case of mpox was reported in Gansu Province in November 2023. Since January 1, 2022, China, including Taiwan, Macau, and Hong Kong, has ranked 10th globally in the number of confirmed mpox cases reported by the WHO [5]. The number of confirmed mpox cases in China (n=2031) was lower than that in the United States (n=31,800), Brazil (n=10,967), Spain (n=7898), France (n=4195), Colombia (n=4090), Mexico (n=4081), the United Kingdom (n=3892), Germany (n=3816), and Peru (n=3812). Therefore, mpox has emerged as a major public health emergency in mainland China, necessitating the implementation of effective prevention and control measures to minimize its impact. This is crucial for safeguarding public health and safety, as well as maintaining a stable social environment.

From June to November, the number of confirmed cases and attack rates of mpox exhibited spatiotemporal heterogeneity. Through local spatial autocorrelation analysis, the geographic locations and patterns of spatial clustering at the provincial level of mpox attack rates within the study areas were identified. The Getis-Ord Gi^*^ statistics identified hot spots with high mpox attack rates in Beijing, Tianjin, Shanghai, Jiangsu, and Hainan provinces. Beijing and Tianjin exhibited consistently high mpox attack rates from June to October. The local Moran *I* statistic was used to validate and supplement the Getis-Ord Gi^*^ analysis. Local Moran *I* statistics identified HH clustering in Guangdong, Beijing, and Tianjin, indicating that areas with high mpox attack rates were clustered among neighboring provinces. Among these provinces, Guangdong consistently exhibited HH clustering in terms of spatial patterns of mpox attack rates from June to November, while Beijing and Tianjin were identified as HH clusters from July to September. In June, October, and November, Beijing was identified as an HL outlier, suggesting that the areas with high mpox attack rates in Beijing were surrounded by other areas with low values. Similarly, Tianjin was recognized as an LH outlier in June and November, indicating that the areas with low mpox attack rates in Tianjin were surrounded by other areas with high values. This finding aligns with the identification of Beijing as an HL outlier in June and November, emphasizing the consistently high mpox attack rates in Beijing. Further research is necessary to comprehend the reasons behind these high attack rates in Beijing, with possible reasons including Beijing’s status as the capital and most densely populated area in China, along with the presence of a large floating population that may contribute to virus transmission. Therefore, the results of the local Moran *I* highlighted an HH clustering in Guangdong Province that had been overlooked in the Getis-Ord Gi^*^ analysis and verified the result that Beijing and Tianjin were consistent hot spots identified by the Getis-Ord Gi^*^ analysis. Additionally, Sichuan was identified as an HL outlier in July, August, and October, whereas Jiangsu was identified as an LH outlier in October, suggesting that the surrounding Shanghai and Zhejiang provinces had high mpox attack rates. Therefore, all the hot spots and HH clustering of mpox attack rates identified through local spatial autocorrelation analysis should be considered key areas for precision prevention and control of mpox. Specifically, the Guangdong, Beijing, and Tianjin provinces, which consistently exhibited high-value clustering, should be given high priority for mpox prevention and control. These findings provide geographically precise and visual evidence to assist in identifying key areas for the targeted prevention and control of mpox.

The GWR model was used to analyze the spatially varying relationships between independent and dependent variables while considering spatial autocorrelation [34]. It used the weighted least squares method to estimate varying parameters locally, allowing for varied spatial relationships across different geographical regions. This localized approach facilitated the identification of spatial heterogeneity, revealing trends and distribution patterns of the spatial relationship between independent and dependent variables within specific spatial extents [30,34]. In contrast, the OLS model estimated parameters using the OLS method, assuming that spatial relationships in the model were constant across space [30]. However, this global parameter estimation with constant parameters overlooks spatial heterogeneity in the data and fails to capture spatial variation trends [30]. In this study, the GWR model revealed a positive association and spatial heterogeneity between the cumulative mpox attack rates and the PUP, PCGDP, PCDI, and PCCE. This finding aligns with the results obtained from the application of the Getis-Ord Gi^*^ statistics, which identified hot spots in the Beijing, Tianjin, Shanghai, Jiangsu, and Hainan provinces. Furthermore, the results of the Local Moran *I* statistics also supported this finding, indicating HH clustering in Guangdong, Beijing, and Tianjin and HL outliers in Sichuan. These areas, which were identified as key regions for the precise prevention and control of mpox through local autocorrelation analysis, are characterized by robust economic development [28].

### Limitations

This study had 2 limitations. First, the spatial analysis was conducted at the provincial level, and further studies should consider smaller geographic units for more accurate evidence. Second, this study focused solely on the relationship between cumulative mpox attack rates and sociodemographic and socioeconomic factors; future studies should also consider natural and environmental risk factors.

### Conclusions

A total of 1610 locally confirmed mpox cases were reported in 30 provinces in mainland China from June to November 2023, resulting in an attack rate of 11.40 per 10 million people. The hot spots and HH clustering of mpox attack rates should be considered as key areas for precision prevention and control of mpox. Specifically, Guangdong, Beijing, and Tianjin should be given high priority for mpox prevention and control. These findings provide geographically precise and visual evidence for identifying key areas for targeted prevention and control.

## Appendix

Multimedia Appendix 1 Supplementary Tables S1-S10.

Multimedia Appendix 2 Spatial distribution of the study areas (A) and administrative divisions (B).

Multimedia Appendix 3 Spatial distribution of the cumulative confirmed cases (A) and attack rates (B) of mpox.

Multimedia Appendix 4 Higher resolution version of Figure 2.

Multimedia Appendix 5 Higher resolution version of Figure 4.

Multimedia Appendix 6 Spatial distribution of the provincial-level values of explanatory variables.

Multimedia Appendix 7 Correlation analysis between the cumulative mpox attack rates and explanatory variables.

Multimedia Appendix 8 Higher resolution version of Figure 5.

## Abbreviations

AR: aging rate
GWR: geographically weighted regression
HH: high-high
HL: high-low
LH: low-high
NPGR: natural population growth rate
OLS: ordinary least square
PCCE: per capita consumption expenditure
PCCEH: per capita consumption expenditure for health care
PCDI: per capita disposable income
PCGDP: per capita gross domestic product
PCRA: per capita road area
PUP: proportion of the urban population
WHO: World Health Organization

## Data Availability

The data sets used or analyzed during the current study are available from the corresponding author on reasonable request.
