# Supplementary material for: Spatiotemporal Epidemiological Trends of Mpox in Mainland China: Spatiotemporal Ecological Comparison Study
Source: JMIR Public Health Surveill. 2024 Jun 19;10:e57807. doi: 10.2196/57807 (PMC11229661; doi:10.2196/57807)
Supplement: Multimedia Appendix 1 [file publichealth_v10i1e57807_app1.docx]

**Spatio-Temporal Epidemiological Trends of Mpox in Mainland China**

**Supplementary Appendix**

**TABLE OF CONTENTS**

[Table S1. Study aeas for spatiotemporal analysis of MPX at the province level in mainland China 2](#_Toc162707628)

[Table S2. Confirmed cases and attack rates of MPX from June 1 to November 30, 2023 at the province level in mainland China 3](#_Toc162707629)

[Table S3. The results of global Moran’s *I* analysis of attack rates of MPX at the province level in mainland China from June to November, 2023 5](#_Toc162707630)

[Table S4. Descriptive statistics of the explanatory variables 6](#_Toc162707631)

[Table S5. The province-level values of the explanatory variables 7](#_Toc162707632)

[Table S6. The result of OLS regression model between the cumulative MPX attack rates and explanatory variables 9](#_Toc162707633)

[Table S7. Descriptive statistics of the coefficients values of the explanatory variables in the GWR regression model 11](#_Toc162707634)

[Table S8. The coefficients values of the explanatory variables in the GWR regression model at the province level in mainland China 12](#_Toc162707635)

[Table S9. Descriptive statistics of the local *R*^2^ values of the explanatory variables in the GWR regression model 14](#_Toc162707636)

[Table S10. The local *R*^2^ values of the explanatory variables in the GWR regression model at the province level in mainland China 15](#_Toc162707637)

**Table S1.** Study aeas for spatiotemporal analysis of MPX at the province level in mainland China

| **Number** | **Province** | **Administrative division** | **Number** | **Province** | **Administrative division** |
| --- | --- | --- | --- | --- | --- |
| 1 | Beijing | North China | 17 | Hubei | Central South China |
| 2 | Tianjin | North China | 18 | Hunan | Central South China |
| 3 | Hebei | North China | 19 | Guangdong | Central South China |
| 4 | Shanxi | North China | 20 | Guangxi | Central South China |
| 5 | Inner Mongolia | North China | 21 | Hainan | Central South China |
| 6 | Liaoning | Northeast China | 22 | Chongqing | Southwest China |
| 7 | Jilin | Northeast China | 23 | Sichuan | Southwest China |
| 8 | Heilongjiang | Northeast China | 24 | Guizhou | Southwest China |
| 9 | Shanghai | East China | 25 | Yunnan | Southwest China |
| 10 | Jiangsu | East China | 26 | Xizang | Southwest China |
| 11 | Zhejiang | East China | 27 | Shaanxi | Northwest China |
| 12 | Anhui | East China | 28 | Gansu | Northwest China |
| 13 | Fujian | East China | 29 | Qinghai | Northwest China |
| 14 | Jiangxi | East China | 30 | Ningxia | Northwest China |
| 15 | Shandong | East China | 31 | Xinjiang | Northwest China |
| 16 | Henan | Central South China | - | - | - |

**Table** **S2.** Confirmed cases and attack rates of MPX from June 1 to November 30, 2023 at the province level in mainland China

| **Province** | **Confirmed cases** | | | | | | |  | **Attack rates (1/10,000,000)** | | | | | | |
| --- | --- | --- | --- | --- | --- | --- | --- | --- | --- | --- | --- | --- | --- | --- | --- |
|  | **June** | **July** | **August** | **September** | **October** | **November** | **Sum** |  | **June** | **July** | **August** | **September** | **October** | **November** | **Sum** |
| Beijing | 45 | 81 | 54 | 42 | 13 | 10 | 245 |  | 20.60 | 37.09 | 24.73 | 19.23 | 5.95 | 4.58 | 112.18 |
| Tianjin | 0 | 17 | 13 | 7 | 2 | 0 | 39 |  | 0.00 | 12.47 | 9.54 | 5.14 | 1.47 | 0.00 | 28.61 |
| Hebei | 0 | 0 | 7 | 8 | 1 | 0 | 16 |  | 0.00 | 0.00 | 0.94 | 1.08 | 0.13 | 0.00 | 2.16 |
| Shanxi | 0 | 1 | 4 | 3 | 0 | 0 | 8 |  | 0.00 | 0.29 | 1.15 | 0.86 | 0.00 | 0.00 | 2.30 |
| Inner Mongolia | 0 | 1 | 3 | 1 | 1 | 1 | 7 |  | 0.00 | 0.42 | 1.25 | 0.42 | 0.42 | 0.42 | 2.92 |
| Liaoning | 0 | 12 | 2 | 11 | 1 | 3 | 29 |  | 0.00 | 2.86 | 0.48 | 2.62 | 0.24 | 0.71 | 6.91 |
| Jilin | 0 | 9 | 5 | 4 | 0 | 3 | 21 |  | 0.00 | 3.83 | 2.13 | 1.70 | 0.00 | 1.28 | 8.94 |
| Heilongjiang | 0 | 0 | 6 | 5 | 4 | 0 | 15 |  | 0.00 | 0.00 | 1.94 | 1.61 | 1.29 | 0.00 | 4.84 |
| Shanghai | 0 | 25 | 32 | 6 | 16 | 8 | 87 |  | 0.00 | 10.10 | 12.93 | 2.42 | 6.46 | 3.23 | 35.15 |
| Jiangsu | 8 | 31 | 29 | 32 | 7 | 7 | 114 |  | 0.94 | 3.64 | 3.41 | 3.76 | 0.82 | 0.82 | 13.39 |
| Zhejiang | 1 | 40 | 77 | 38 | 15 | 5 | 176 |  | 0.15 | 6.08 | 11.71 | 5.78 | 2.28 | 0.76 | 26.76 |
| Anhui | 0 | 19 | 12 | 4 | 0 | 4 | 39 |  | 0.00 | 3.10 | 1.96 | 0.65 | 0.00 | 0.65 | 6.37 |
| Fujian | 0 | 2 | 7 | 6 | 4 | 4 | 23 |  | 0.00 | 0.48 | 1.67 | 1.43 | 0.96 | 0.96 | 5.49 |
| Jiangxi | 0 | 0 | 1 | 3 | 1 | 1 | 6 |  | 0.00 | 0.00 | 0.22 | 0.66 | 0.22 | 0.22 | 1.33 |
| Shandong | 2 | 12 | 10 | 9 | 4 | 1 | 38 |  | 0.20 | 1.18 | 0.98 | 0.89 | 0.39 | 0.10 | 3.74 |
| Henan | 0 | 14 | 13 | 6 | 8 | 1 | 42 |  | 0.00 | 1.42 | 1.32 | 0.61 | 0.81 | 0.10 | 4.25 |
| Hubei | 2 | 17 | 11 | 12 | 6 | 2 | 50 |  | 0.34 | 2.91 | 1.88 | 2.05 | 1.03 | 0.34 | 8.56 |
| Hunan | 0 | 33 | 13 | 11 | 5 | 5 | 67 |  | 0.00 | 5.00 | 1.97 | 1.67 | 0.76 | 0.76 | 10.15 |
| Guangdong | 48 | 115 | 95 | 40 | 17 | 12 | 327 |  | 3.79 | 9.09 | 7.51 | 3.16 | 1.34 | 0.95 | 25.84 |
| Guangxi | 0 | 1 | 21 | 16 | 6 | 3 | 47 |  | 0.00 | 0.20 | 4.16 | 3.17 | 1.19 | 0.59 | 9.31 |
| Hainan | 0 | 0 | 0 | 1 | 0 | 3 | 4 |  | 0.00 | 0.00 | 0.00 | 0.97 | 0.00 | 2.92 | 3.89 |
| Chongqing | 0 | 2 | 12 | 12 | 2 | 1 | 29 |  | 0.00 | 0.62 | 3.73 | 3.73 | 0.62 | 0.31 | 9.03 |
| Sichuan | 0 | 49 | 54 | 13 | 12 | 4 | 132 |  | 0.00 | 5.85 | 6.45 | 1.55 | 1.43 | 0.48 | 15.76 |
| Guizhou | 0 | 1 | 2 | 1 | 0 | 1 | 5 |  | 0.00 | 0.26 | 0.52 | 0.26 | 0.00 | 0.26 | 1.30 |
| Yunnan | 0 | 4 | 11 | 3 | 2 | 0 | 20 |  | 0.00 | 0.85 | 2.34 | 0.64 | 0.43 | 0.00 | 4.26 |
| Xizang | 0 | 0 | 0 | 0 | 0 | 0 | 0 |  | 0.00 | 0.00 | 0.00 | 0.00 | 0.00 | 0.00 | 0.00 |
| Shaanxi | 0 | 4 | 7 | 9 | 0 | 0 | 20 |  | 0.00 | 1.01 | 1.77 | 2.28 | 0.00 | 0.00 | 5.06 |
| Gansu | 0 | 0 | 0 | 0 | 0 | 1 | 1 |  | 0.00 | 0.00 | 0.00 | 0.00 | 0.00 | 0.40 | 0.40 |
| Qinghai | 0 | 1 | 0 | 0 | 0 | 0 | 1 |  | 0.00 | 1.68 | 0.00 | 0.00 | 0.00 | 0.00 | 1.68 |
| Ningxia | 0 | 0 | 0 | 1 | 0 | 0 | 1 |  | 0.00 | 0.00 | 0.00 | 1.37 | 0.00 | 0.00 | 1.37 |
| Xinjiang | 0 | 0 | 0 | 1 | 0 | 0 | 1 |  | 0.00 | 0.00 | 0.00 | 0.39 | 0.00 | 0.00 | 0.39 |
| Total | 106 | 491 | 501 | 305 | 127 | 80 | 1610 |  | 0.75 | 3.48 | 3.55 | 2.16 | 0.90 | 0.57 | 11.40 |

**Table** **S3.** The results of global Moran’s *I* analysis of attack rates of MPX at the province level in mainland China from June to November, 2023

| **Month** | **Moran’s *I*** | ***z*-value** | ***P*-value** | **Pattern** |
| --- | --- | --- | --- | --- |
| June | -0.0057 | 0.6822 | .50 | Random |
| July | 0.0938 | 1.7251 | .08 | Clustered |
| August | 0.1276 | 1.7260 | .08 | Clustered |
| September | 0.0934 | 1.8450 | .07 | Clustered |
| October | 0.0969 | 1.3871 | .17 | Random |
| November | 0.0510 | 0.8677 | .39 | Random |

**Table** **S4.** Descriptive statistics of the explanatory variables

| **Variable** | **Mean (SD)** | **Min** | ***P*_25_** | **Median** | ***P*_75_** | **Max** |
| --- | --- | --- | --- | --- | --- | --- |
| PUP (%) | 65.00 (10.65) | 37.39 | 58.35 | 63.96 | 70.96 | 89.33 |
| NPGR (‰) | -0.23 (2.91) | -5.75 | -2.01 | -0.28 | 0.77 | 8.76 |
| PIP (%) | 4.60 (5.96) | 0.84 | 2.19 | 3.02 | 4.29 | 34.55 |
| AR (%) | 14.39 (3.33) | 5.90 | 12.12 | 14.69 | 17.01 | 20.02 |
| PCRA (m^2^) | 20.67 (5.53) | 11.82 | 16.08 | 19.68 | 24.48 | 34.43 |
| PCGDP (Yuan) | 84461 (36090) | 44968 | 60724 | 70923 | 96474 | 190313 |
| PCDI (Yuan) | 36583 (13931) | 23273 | 27981 | 30957 | 37560 | 79610 |
| PCCE (Yuan) | 23951 (7686) | 15886 | 18343 | 21708 | 25371 | 46045 |
| PCCEH (Yuan) | 2144 (648) | 727 | 1803 | 2017 | 2351 | 3982 |

**Table** **S5.** The province-level values of the explanatory variables

| **Province** | **PUP**  **(%)** | **NPGR**  **(‰)** | **PIP**  **(%)** | **AR**  **(%)** | **PCRA**  **(m^2^)** | **PCGDP**  **(Yuan)** | **PCDI**  **(Yuan)** | **PCCE**  **(Yuan)** | **PCCEH**  **(Yuan)** |
| --- | --- | --- | --- | --- | --- | --- | --- | --- | --- |
| Beijing | 87.57 | -0.05 | 0.84 | 15.12 | - | 190313 | 77414.5 | 42683.2 | 3981.5 |
| Tianjin | 85.11 | -1.68 | 1.65 | 17.01 | - | 119235 | 48976.1 | 31323.7 | 3555.5 |
| Hebei | 61.65 | -1.71 | 2.18 | 15.64 | 25.46 | 56995 | 30867.0 | 20890.3 | 2017.3 |
| Shanxi | 63.96 | -0.98 | 1.73 | 14.48 | 17.00 | 73675 | 29178.2 | 17536.7 | 1943.6 |
| Inner Mongolia | 68.60 | -2.25 | 3.75 | 14.69 | 33.97 | 96474 | 35920.6 | 22298.4 | 2262.7 |
| Liaoning | 73.00 | -4.96 | 1.49 | 20.02 | 16.12 | 68775 | 36088.8 | 22603.7 | 2192.3 |
| Jilin | 63.72 | -4.07 | 1.82 | 17.75 | 17.77 | 55347 | 27974.5 | 17897.5 | 2067.9 |
| Heilongjiang | 66.21 | -5.75 | 2.48 | 17.82 | 14.53 | 51096 | 28345.5 | 20411.9 | 2524.9 |
| Shanghai | 89.33 | -1.61 | 1.66 | 18.67 | - | 179907 | 79609.8 | 46045.4 | 3616.5 |
| Jiangsu | 74.42 | -1.81 | 2.79 | 17.87 | 22.87 | 144390 | 49861.7 | 32848.1 | 2564.0 |
| Zhejiang | 73.38 | 0.04 | 3.71 | 14.91 | 25.63 | 118496 | 60302.5 | 38971.1 | 2533.5 |
| Anhui | 60.15 | -0.93 | 5.05 | 15.79 | 25.15 | 73603 | 32745.2 | 22541.9 | 1749.6 |
| Fujian | 70.11 | 0.55 | 2.90 | 12.19 | 19.86 | 126829 | 43117.7 | 30041.7 | 1904.7 |
| Jiangxi | 62.07 | 0.25 | 2.37 | 13.03 | 25.27 | 70923 | 32418.7 | 21707.9 | 1856.0 |
| Shandong | 64.54 | -0.93 | 4.28 | 16.72 | 22.83 | 86003 | 37560.1 | 22640.4 | 2014.6 |
| Henan | 57.07 | -0.08 | 3.20 | 14.55 | 18.68 | 62106 | 28222.4 | 19019.5 | 1920.1 |
| Hubei | 64.67 | -2.01 | 3.02 | 16.29 | 19.50 | 92059 | 32913.6 | 24827.8 | 2299.9 |
| Hunan | 60.31 | -2.31 | 2.41 | 16.12 | 14.83 | 73598 | 34036.0 | 24082.7 | 2295.7 |
| Guangdong | 74.79 | 3.33 | 2.19 | 9.60 | 14.92 | 101905 | 47064.6 | 32168.7 | 1783.0 |
| Guangxi | 55.65 | 1.43 | 2.96 | 13.13 | 21.79 | 52164 | 27980.7 | 18342.8 | 1803.3 |
| Hainan | 61.49 | 2.44 | 3.66 | 11.29 | 34.43 | 66602 | 30956.6 | 21500.4 | 1372.9 |
| Chongqing | 70.96 | -2.11 | 2.20 | 18.30 | 11.82 | 90663 | 35665.9 | 25371.1 | 2350.5 |
| Sichuan | 58.35 | -2.65 | 4.29 | 18.12 | 15.10 | 67777 | 30679.2 | 22301.9 | 2105.4 |
| Guizhou | 54.81 | 3.71 | 7.98 | 12.12 | 21.25 | 52321 | 25508.2 | 17938.7 | 1373.7 |
| Yunnan | 51.72 | -0.07 | 5.56 | 11.67 | 19.26 | 61716 | 26936.8 | 18950.8 | 1825.2 |
| Xizang | 37.39 | 8.76 | 34.55 | 5.90 | 17.16 | 58438 | 26674.8 | 15885.6 | 726.5 |
| Shaanxi | 64.02 | -0.28 | 3.35 | 14.68 | 16.07 | 82864 | 30115.8 | 19848.4 | 2400.3 |
| Gansu | 54.19 | -0.04 | 9.05 | 13.43 | 15.49 | 44968 | 23273.1 | 17489.4 | 1612.6 |
| Qinghai | 61.43 | 3.37 | 9.69 | 10.22 | 23.24 | 60724 | 27000.0 | 17260.8 | 1768.3 |
| Ningxia | 66.34 | 4.41 | 5.96 | 10.43 | 24.64 | 69781 | 29599.3 | 19136.3 | 2067.2 |
| Xinjiang | 57.89 | 0.77 | 3.91 | 8.39 | 23.98 | 68552 | 27062.7 | 17927.1 | 1968.5 |

**Table** **S6.** The result of OLS regression model between the cumulative MPX attack rates and explanatory variables

| **Independent variables** | ***β*** | **$S_{\bar{x}}$** | ***t*-value** | ***P*-value** | ***R*^2^** | **AICc** |
| --- | --- | --- | --- | --- | --- | --- |
| PUP |  |  |  |  |  |  |
| Intercept | -70.2391 | 18.1478 | -2.1805 | .04 | - |  |
| PUP | 1.2605 | 0.2757 | 2.4041 | .02 | 0.4190 | 264.9909 |
| NPGR |  |  |  |  |  |  |
| Intercept | 11.5565 | 3.7884 | 3.0505 | .005 | - |  |
| NPGR | -0.5663 | 1.3186 | -0.4295 | .67 | 0.0063 | 281.6248 |
| PIP |  |  |  |  |  |  |
| Intercept | 15.4758 | 4.6807 | 3.3063 | .003 | - |  |
| PIP | -0.8229 | 0.6283 | -1.3097 | .20 | 0.0558 | 280.0398 |
| AR |  |  |  |  |  |  |
| Intercept | -6.2152 | 16.6997 | -0.3722 | .71 | - |  |
| AR | 1.2445 | 1.1318 | 1.0996 | .28 | 0.0400 | 280.5551 |
| PCRA |  |  |  |  |  |  |
| Intercept | 11.5336 | 5.0737 | 2.2732 | .03 | - |  |
| PCRA | -0.2360 | 0.2375 | -0.9937 | .33 | 0.0366 | 191.8875 |
| PCGDP |  |  |  |  |  |  |
| Intercept | -25.0882 | 6.3775 | -2.1450 | .04 | - |  |
| PCGDP | 0.0004 | 0.0001 | 2.6955 | .01 | 0.5743 | 255.3438 |
| PCDI |  |  |  |  |  |  |
| Intercept | -32.1063 | 6.4166 | -2.3664 | .02 | - |  |
| PCDI | 0.0012 | 0.0002 | 2.8303 | .008 | 0.6469 | 249.5536 |
| PCCE |  |  |  |  |  |  |
| Intercept | -36.5956 | 8.3629 | -2.3772 | .02 | - |  |
| PCCE | 0.0020 | 0.0003 | 2.7452 | .01 | 0.5583 | 256.4901 |
| PCCEH |  |  |  |  |  |  |
| Intercept | -38.6876 | 9.0084 | -2.1615 | .04 | - |  |
| PCCEH | 0.0235 | 0.0040 | 2.5924 | .01 | 0.5400 | 257.7505 |

**Table** **S7.** Descriptive statistics of the coefficients values of the explanatory variables in the GWR regression model

| **Independent variables** | **Min** | ***P*_25_** | **Median** | ***P*_75_** | **Max** | ***R*^2^** | **AICc** |
| --- | --- | --- | --- | --- | --- | --- | --- |
| PUP | 1.0000 | 1.2559 | 1.4513 | 1.6897 | 2.0349 | 0.5334 | 262.5318 |
| PCGDP | 0.00025 | 0.00034 | 0.00046 | 0.00056 | 0.00071 | 0.7653 | 251.1139 |
| PCDI | 0.0008 | 0.0010 | 0.0012 | 0.0015 | 0.0018 | 0.8092 | 239.4085 |
| PCCE | 0.0013 | 0.0016 | 0.0020 | 0.0027 | 0.0034 | 0.7640 | 248.7289 |
| PCCEH | 0.0181 | 0.0226 | 0.0266 | 0.0303 | 0.0345 | 0.6652 | 254.0077 |

**Table** **S8.** The coefficients values of the explanatory variables in the GWR regression model at the province level in mainland China

| **Province** | **PUP** | **PCGDP** | **PCDI** | **PCCE** | **PCCEH** |
| --- | --- | --- | --- | --- | --- |
| Beijing | 1.7425 | 0.00063 | 0.0016 | 0.0029 | 0.0315 |
| Tianjin | 1.7311 | 0.00060 | 0.0015 | 0.0027 | 0.0311 |
| Hebei | 1.7204 | 0.00062 | 0.0015 | 0.0028 | 0.0311 |
| Shanxi | 1.5964 | 0.00059 | 0.0015 | 0.0027 | 0.0295 |
| Inner Mongolia | 1.8095 | 0.00071 | 0.0018 | 0.0034 | 0.0332 |
| Liaoning | 1.8388 | 0.00055 | 0.0015 | 0.0027 | 0.0321 |
| Jilin | 1.9205 | 0.00052 | 0.0015 | 0.0026 | 0.0330 |
| Heilongjiang | 2.0349 | 0.00054 | 0.0015 | 0.0029 | 0.0345 |
| Shanghai | 1.5556 | 0.00038 | 0.0010 | 0.0018 | 0.0266 |
| Jiangsu | 1.5832 | 0.00043 | 0.0012 | 0.0020 | 0.0276 |
| Zhejiang | 1.4915 | 0.00034 | 0.0010 | 0.0017 | 0.0254 |
| Anhui | 1.5260 | 0.00042 | 0.0011 | 0.0019 | 0.0268 |
| Fujian | 1.3920 | 0.00029 | 0.0009 | 0.0015 | 0.0234 |
| Jiangxi | 1.3979 | 0.00032 | 0.0010 | 0.0016 | 0.0241 |
| Shandong | 1.6590 | 0.00052 | 0.0013 | 0.0024 | 0.0295 |
| Henan | 1.5230 | 0.00049 | 0.0013 | 0.0022 | 0.0276 |
| Hubei | 1.4214 | 0.00041 | 0.0012 | 0.0019 | 0.0256 |
| Hunan | 1.3273 | 0.00032 | 0.0010 | 0.0016 | 0.0235 |
| Guangdong | 1.2622 | 0.00025 | 0.0009 | 0.0014 | 0.0213 |
| Guangxi | 1.1800 | 0.00025 | 0.0009 | 0.0014 | 0.0205 |
| Hainan | 1.1103 | 0.00022^a^ | 0.0008 | 0.0013 | 0.0183 |
| Chongqing | 1.2967 | 0.00037 | 0.0011 | 0.0019 | 0.0239 |
| Sichuan | 1.1534 | 0.00035 | 0.0012 | 0.0019 | 0.0217 |
| Guizhou | 1.1964 | 0.00030 | 0.0010 | 0.0016 | 0.0216 |
| Yunnan | 1.0030 | 0.00026^a^ | 0.0010 | 0.0015 | 0.0181 |
| Xizang | 0.6417^a^ | 0.00022^a^ | 0.0015 | 0.0017^a^ | 0.0103^a^ |
| Shaanxi | 1.4513 | 0.00053 | 0.0014 | 0.0025 | 0.0273 |
| Gansu | 1.2496 | 0.00048 | 0.0015 | 0.0027 | 0.0244 |
| Qinghai | 1.0000 | 0.00036^a^ | 0.0014 | 0.0022 | 0.0188 |
| Ningxia | 1.4259 | 0.00056 | 0.0015 | 0.0027 | 0.0274 |
| Xinjiang | 0.6223^a^ | 0.00002^a^ | 0.0014^a^ | 0.0012^a^ | 0.0090^a^ |

^a^ represents no statistical significance.

**Table** **S9.** Descriptive statistics of the local *R*^2^ values of the explanatory variables in the GWR regression model

| **Independent variables** | **Min** | ***P*_25_** | **Median** | ***P*_75_** | **Max** |
| --- | --- | --- | --- | --- | --- |
| PUP | 0.3484 | 0.4273 | 0.4627 | 0.4827 | 0.5176 |
| PCGDP | 0.3976 | 0.4684 | 0.5698 | 0.6706 | 0.7768 |
| PCDI | 0.5869 | 0.5997 | 0.6422 | 0.7106 | 0.7939 |
| PCCE | 0.5016 | 0.5297 | 0.5587 | 0.6291 | 0.7344 |
| PCCEH | 0.4422 | 0.5244 | 0.5927 | 0.6336 | 0.6662 |

**Table** **S10.** The local *R*^2^ values of the explanatory variables in the GWR regression model at the province level in mainland China

| **Province** | **PUP** | **PCGDP** | **PCDI** | **PCCE** | **PCCEH** |
| --- | --- | --- | --- | --- | --- |
| Beijing | 0.4870 | 0.7045 | 0.7211 | 0.6393 | 0.6470 |
| Tianjin | 0.4867 | 0.6799 | 0.7008 | 0.6140 | 0.6424 |
| Hebei | 0.4846 | 0.6960 | 0.7140 | 0.6302 | 0.6431 |
| Shanxi | 0.4674 | 0.6854 | 0.7130 | 0.6280 | 0.6239 |
| Inner Mongolia | 0.4920 | 0.7768 | 0.7939 | 0.7344 | 0.6662 |
| Liaoning | 0.4990 | 0.6551 | 0.6829 | 0.5967 | 0.6504 |
| Jilin | 0.5067 | 0.6592 | 0.6844 | 0.6050 | 0.6538 |
| Heilongjiang | 0.5176 | 0.7063 | 0.7274 | 0.6604 | 0.6618 |
| Shanghai | 0.4772 | 0.5078 | 0.5874 | 0.5016 | 0.5941 |
| Jiangsu | 0.4765 | 0.5430 | 0.6080 | 0.5142 | 0.6040 |
| Zhejiang | 0.4719 | 0.4892 | 0.5869 | 0.5095 | 0.5785 |
| Anhui | 0.4693 | 0.5364 | 0.6110 | 0.5190 | 0.5927 |
| Fujian | 0.4635 | 0.4532 | 0.5912 | 0.5296 | 0.5501 |
| Jiangxi | 0.4582 | 0.4702 | 0.5952 | 0.5198 | 0.5556 |
| Shandong | 0.4809 | 0.6148 | 0.6528 | 0.5575 | 0.6248 |
| Henan | 0.4627 | 0.5967 | 0.6531 | 0.5587 | 0.6002 |
| Hubei | 0.4511 | 0.5334 | 0.6276 | 0.5352 | 0.5722 |
| Hunan | 0.4426 | 0.4632 | 0.6046 | 0.5239 | 0.5399 |
| Guangdong | 0.4456 | 0.4071 | 0.5982 | 0.5470 | 0.5089 |
| Guangxi | 0.4234 | 0.3976 | 0.5985 | 0.5392 | 0.4882 |
| Hainan | 0.4273 | 0.3736^a^ | 0.6116 | 0.5826 | 0.4497 |
| Chongqing | 0.4274 | 0.4989 | 0.6342 | 0.5421 | 0.5419 |
| Sichuan | 0.3940 | 0.4602 | 0.6503 | 0.5625 | 0.5051 |
| Guizhou | 0.4157 | 0.4253 | 0.6089 | 0.5298 | 0.5045 |
| Yunnan | 0.3763 | 0.3596^a^ | 0.5996 | 0.5462 | 0.4422 |
| Xizang | 0.2719^a^ | 0.1641^a^ | 0.5998 | 0.7240^a^ | 0.3124^a^ |
| Shaanxi | 0.4454 | 0.6348 | 0.6973 | 0.6080 | 0.5927 |
| Gansu | 0.3991 | 0.5978 | 0.7521 | 0.6778 | 0.5474 |
| Qinghai | 0.3484 | 0.4360^a^ | 0.7098 | 0.6526 | 0.4571 |
| Ningxia | 0.4359 | 0.6676 | 0.7385 | 0.6591 | 0.5933 |
| Xinjiang | 0.2450^a^ | 0.0062^a^ | 0.5492^a^ | 0.4642^a^ | 0.2535^a^ |

^a^ represents no statistical significance.
