# Supplementary figures and images for: Spatiotemporal Epidemiological Trends of Mpox in Mainland China: Spatiotemporal Ecological Comparison Study
Source: JMIR Public Health Surveill. 2024 Jun 19;10:e57807. doi: 10.2196/57807 (PMC11229661; doi:10.2196/57807)

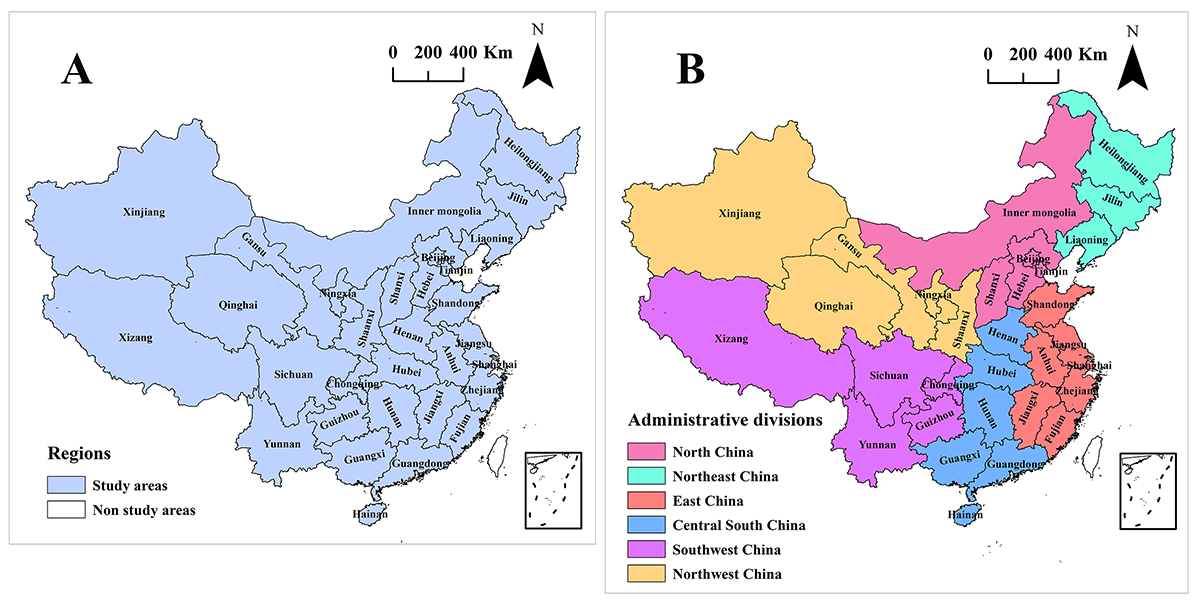

Supplement: Multimedia Appendix 2 [file publichealth_v10i1e57807_app2.png]

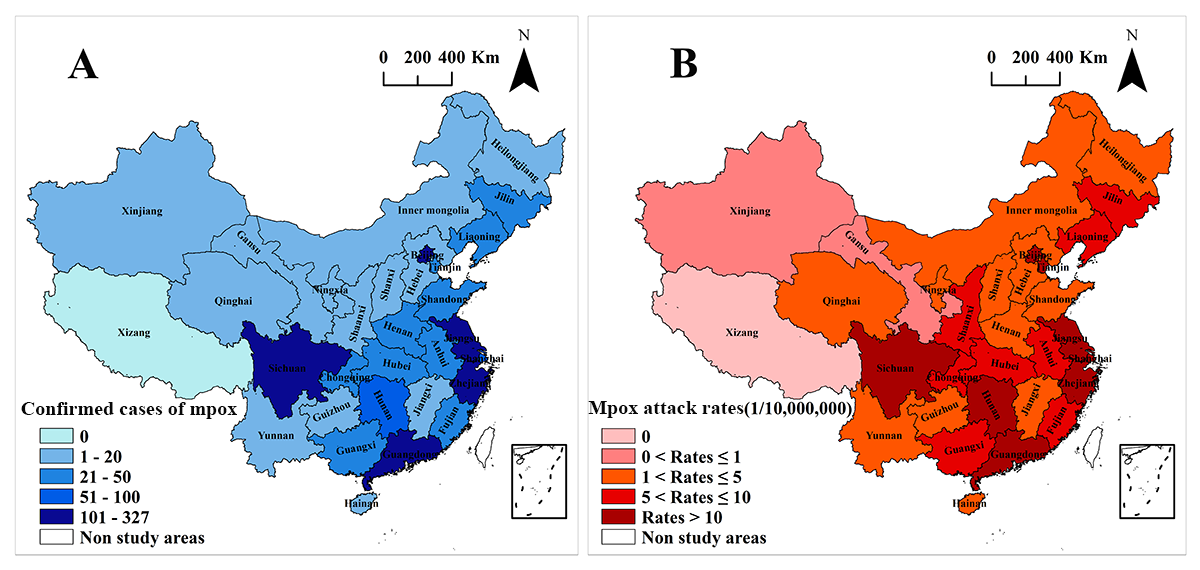

Supplement: Multimedia Appendix 3 [file publichealth_v10i1e57807_app3.png]

A

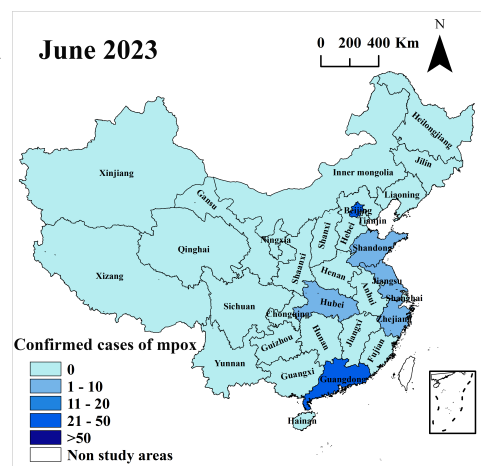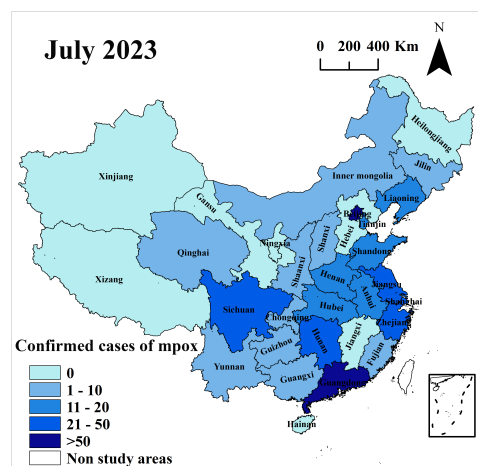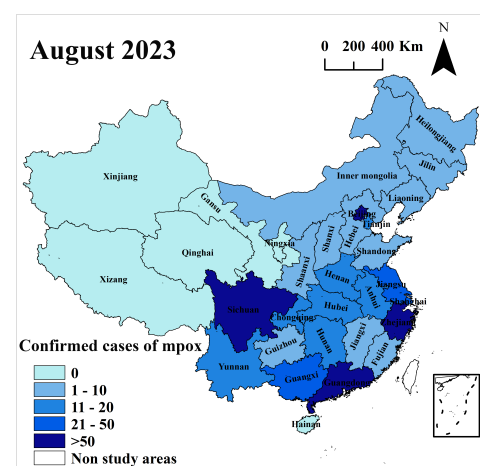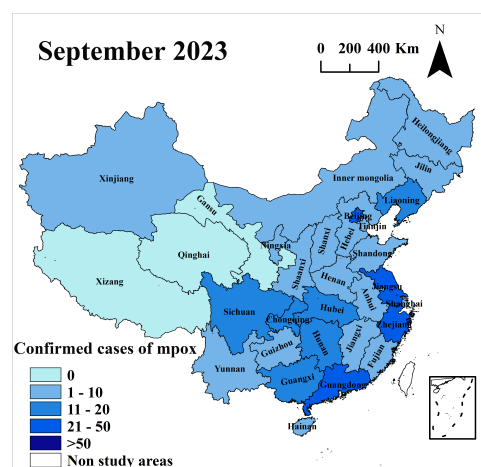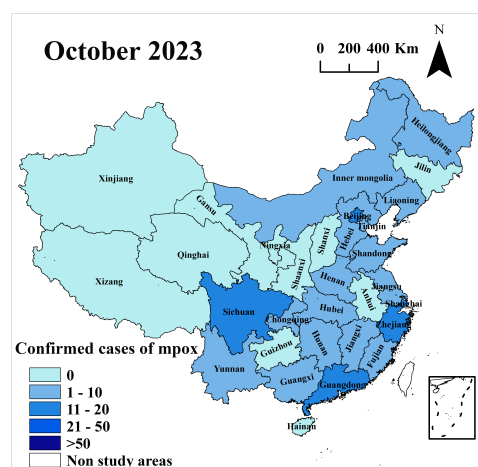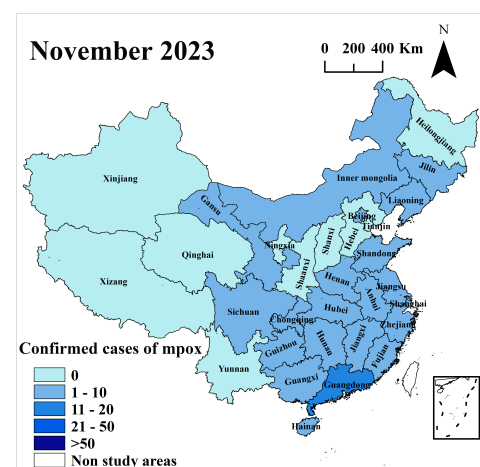

B

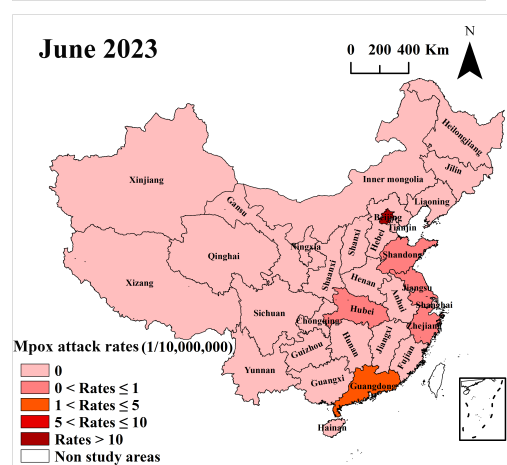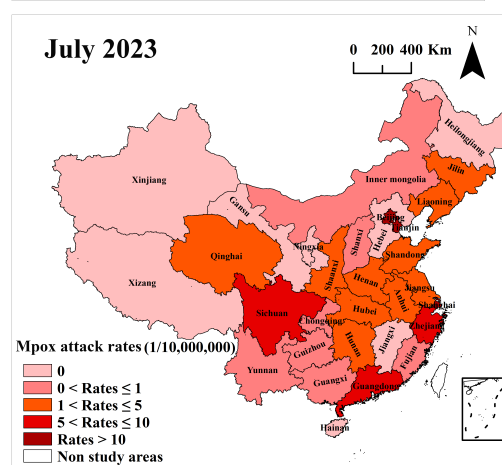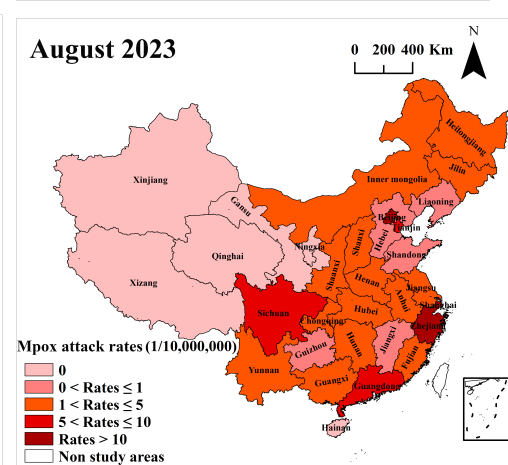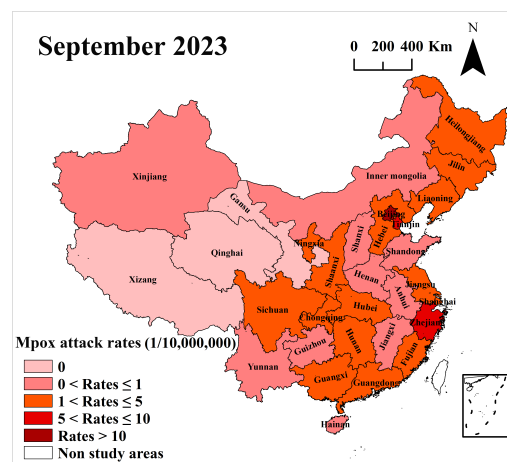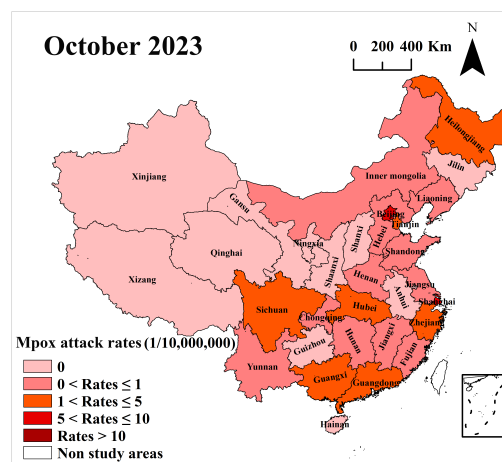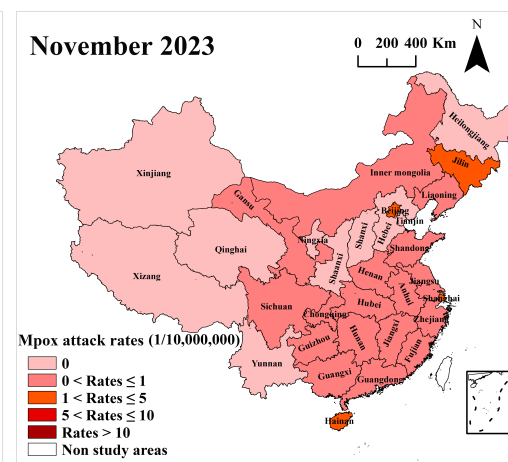

Supplement: Multimedia Appendix 4 [file publichealth_v10i1e57807_app4.pdf]

A

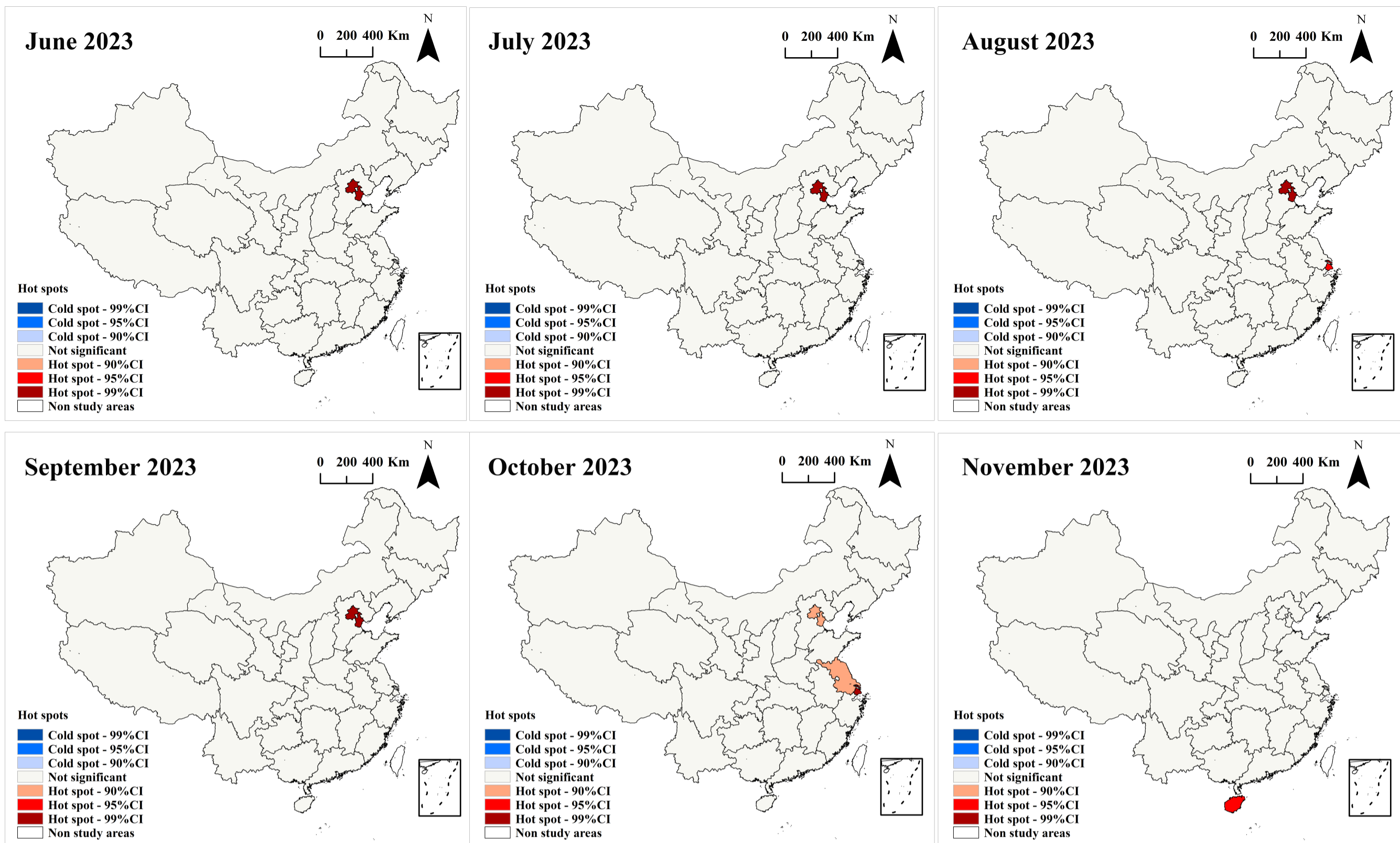

B

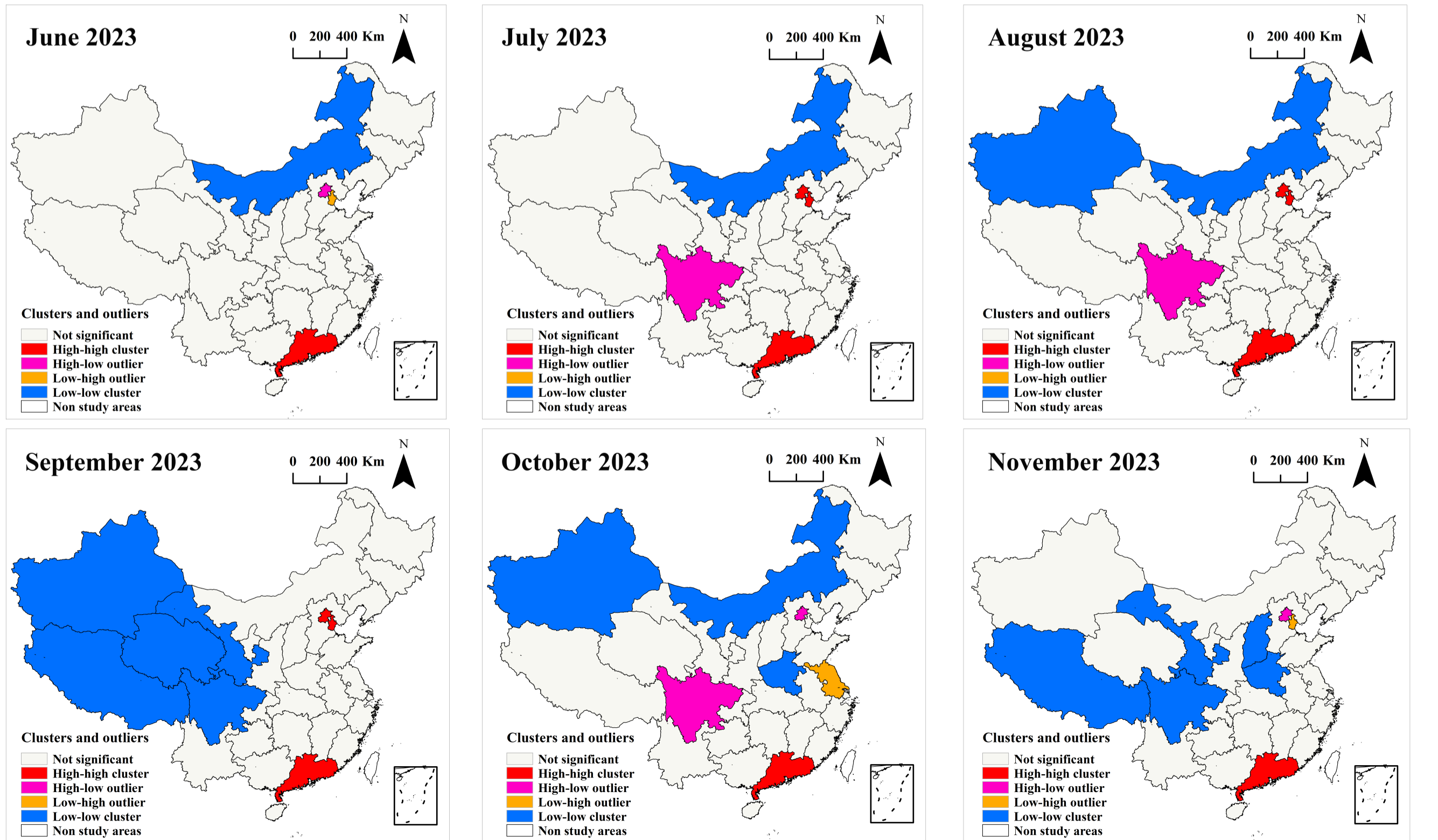

Supplement: Multimedia Appendix 5 [file publichealth_v10i1e57807_app5.pdf]

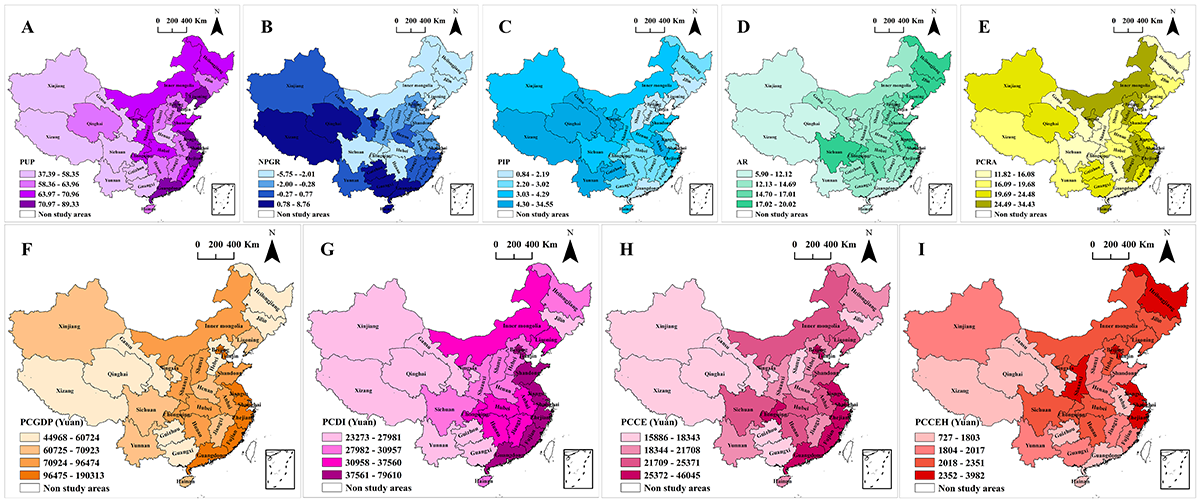

Supplement: Multimedia Appendix 6 [file publichealth_v10i1e57807_app6.png]

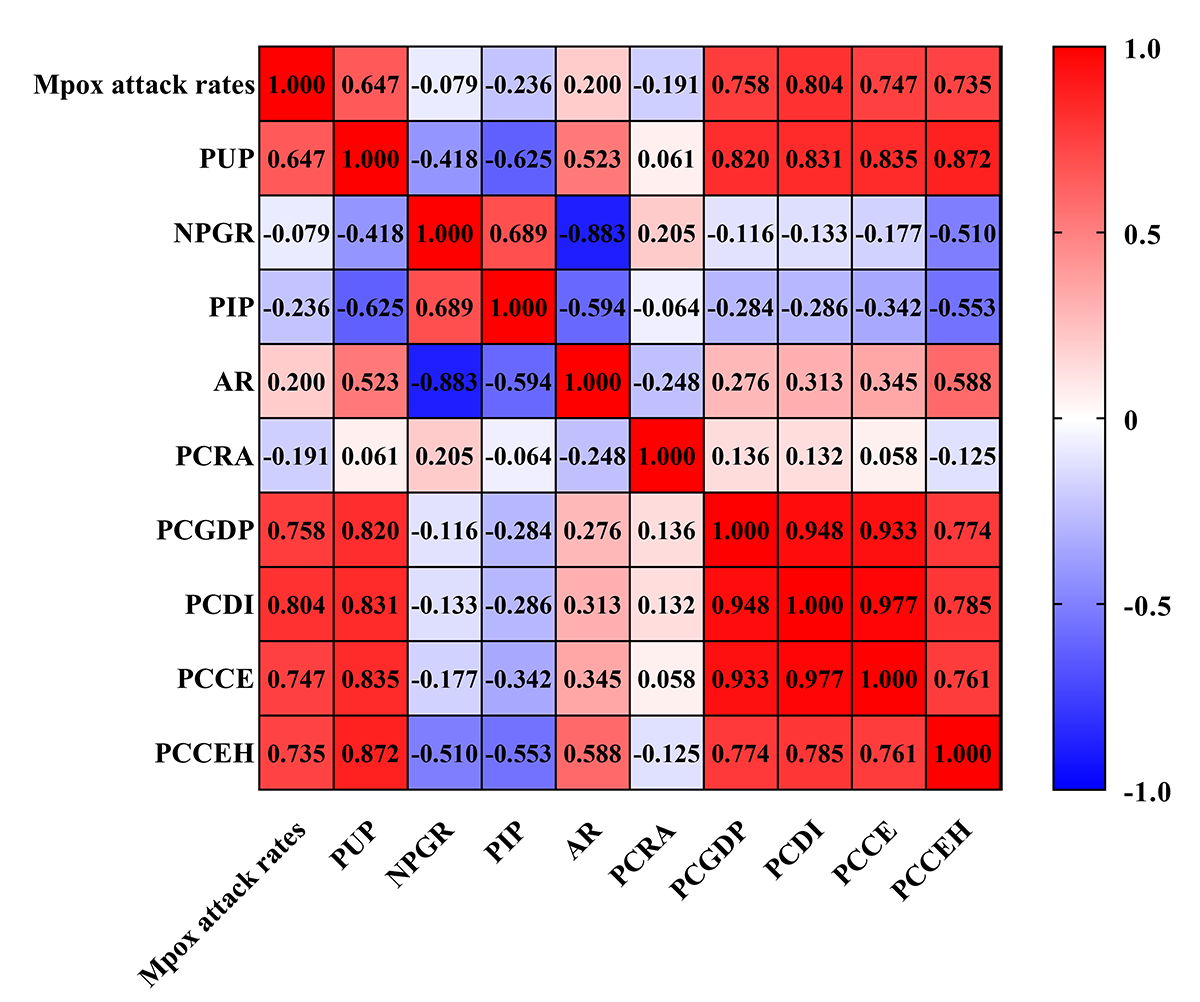

Supplement: Multimedia Appendix 7 [file publichealth_v10i1e57807_app7.png]

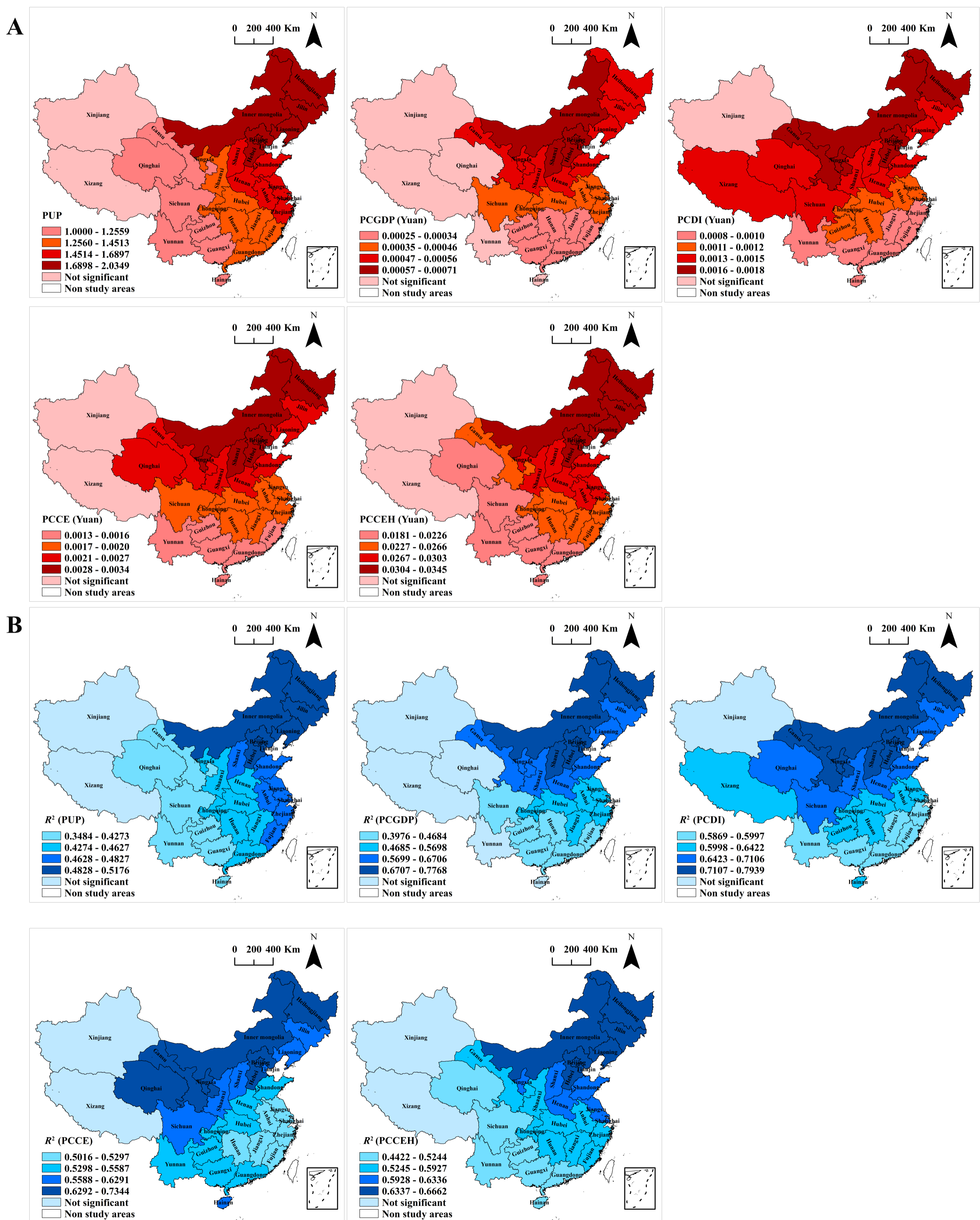

Supplement: Multimedia Appendix 8 [file publichealth_v10i1e57807_app8.pdf]
